# Quantitative Assessment of the Effect of *KCNJ11* Gene Polymorphism on the Risk of Type 2 Diabetes

**DOI:** 10.1371/journal.pone.0093961

**Published:** 2014-04-07

**Authors:** Ling Qiu, Risu Na, Rong Xu, Siyang Wang, Hongguang Sheng, Wanling Wu, Yi Qu

**Affiliations:** 1 Department of Geriatrics, Shanghai Xuhui Central Hospital, Shanghai Clinical Center, Chinese Academy of Sciences, Shanghai, People's Republic of China; 2 Department of Endocrinology, Shanghai Xuhui Central Hospital, Shanghai Clinical Center, Chinese Academy of Sciences, Shanghai, People's Republic of China; 3 Department of Endocrinology, The Ninth People's Hospital Attach to Shanghai Jiao Tong University School of Medicine, Shanghai, People's Republic of China; Children's National Medical Center, Washington, United States of America

## Abstract

To clarify the role of potassium inwardly-rectifying-channel, subfamily-J, member 11 (*KCNJ11*) variation in susceptibility to type 2 diabetes (T2D), we performed a systematic meta-analysis to investigate the association between the *KCNJ11* E23K polymorphism (rs5219) and the T2D in different genetic models. Databases including PubMed, Medline, EMBASE, and ISI Web of Science were searched to identify relevant studies. A total of 48 published studies involving 56,349 T2D cases, 81,800 controls, and 483 family trios were included in this meta-analysis. Overall, the E23K polymorphism was significantly associated with increased T2D risk with per-allele odds ratio (OR) of 1.12 (95% CI: 1.09–1.16; *P*<10^−5^). The summary OR for T2D was 1.09 (95% CI: 1.03–1.14; *P*<10^−5^), and 1.26 (95% CI: 1.17–1.35; *P*<10^−5^), for heterozygous and homozygous, respectively. Similar results were also detected under dominant and recessive genetic models. When stratified by ethnicity, significantly increased risks were found for the polymorphism in Caucasians and East Asians. However, no such associations were detected among Indian and other ethnic populations. Significant associations were also observed in the stratified analyses according to different mean BMI of cases and sample size. Although significant between study heterogeneity was identified, meta-regression analysis suggested that the BMI of controls significantly correlated with the magnitude of the genetic effect. The current meta-analysis demonstrated that a modest but statistically significant effect of the 23K allele of rs5219 polymorphism in susceptibility to T2D. But the contribution of its genetic variants to the epidemic of T2D in Indian and other ethnic populations appears to be relatively low.

## Introduction

Type 2 diabetes (T2D) is a complex metabolic disease resulting from reduced insulin secretion and peripheral insulin resistance. By coupling cell metabolism with membrane potential, adenosine triphosphate-sensitive potassium channel (KATP) play a central role in regulation of insulin secretion in pancreatic-β cells. [1]. The KATP channel is a hetero-octamer of K^+^ inward rectifier Kir6.2 (*KCNJ11*) and regulatory sulfonylurea receptor SUR1 subunits (*ABCC8*) [2]. Mutations in both *KCNJ11* and *ABCC8* cause neonatal diabetes and congenital hyper-insulinemia in humans [3], [4]. In addition, *KCNJ11* gene knock-out mice are characterized by defects in insulin secretion in response to either glucose or tolbutamide [5].

As a candidate gene for T2D in humans, a nonsynonymous E23K variant (rs5219) which results from a G → A transition in codon 23 in the NH_2_-terminal tail of Kir6.2 was identified [6]. With spectacular advance in genotyping method in recent years, larger-scale genetic association study concerning the relationship between the E23K polymorphism and T2D susceptibility has been conducted in various populations. However, inconsistent results have appeared in the literature. Such inconsistence may be due to chance, insufficient power of limited sample size, or bias in study design (e.g., inappropriate control selection). Alternatively, these disparate findings may reflect ethnic diversity (e.g., population stratification) or phenotypic heterogeneity. As a powerful tool for summarizing the results from different studies to estimate the major effect with enhanced precision, meta-analysis has generally been used in quantitative assessment of genetic variation and disease. Here we present the most comprehensive meta-analysis for the effects of E23K polymorphism of *KCNJ11* on T2D risk.

## Materials and Methods

### Literature search strategy and inclusion criteria

To identify eligible literatures, we conducted a computer-based search of PubMed, Medline, EMBASE and ISI Web of Science databases without language restrictions. Studies published before the end of Mar. 2013 on T2D and the E23K polymorphism in the *KCNJ11* gene were retrieved. Search keywords combinations were “potassium inwardly-rectifying-channel, subfamily-J, member 11”, “KCNJ11”, “Kir6.2”, “type 2 diabetes”, “type 2 diabetes mellitus”, “T2D”, “T2DM”, “non-insulin-dependent diabetes mellitus”, “NIDDM”, “polymorphism” or “variation”. The titles and abstracts were read to determine their relevance, and potentially relevant studies were retained for further evaluation. For retrieved articles, the full texts were carefully read to determine whether they meet the purpose of the present meta-analysis. Furthermore, the references of these studies were checked to identify other relevant publications.

Eligible studies should meet following criteria: (1) focusing on the association of the *KCNJ11* E23K polymorphism with T2D risk (2) being case-control or cohort studies (3) diagnosis of T2D patient was confirmed pathologically and (4) providing sufficient data for calculation of odds ratio (OR) with its 95% confidence interval (95% CI) and P-value. The major reasons for exclusion were (1) case-only studies (2) overlapping data (3) review papers. If more than two studies reported the same sample, only the study providing more information or latest published was selected.

### Data extraction

The following information was carefully extracted from all eligible publications: the first author, year of publication, country of origin, ethnicity of subjects, study design, sample size, sex distribution among cases and controls, mean age and body mass index (BMI) of cases and controls, source of control, Hardy–Weinberg equilibrium (HWE) status in controls, genotyping method, number of genotypes in cases and controls. Two authors independently assessed the articles for compliance with the inclusion criteria, and disagreement was followed by discussion until consensus was reached.

### Statistical methods

The association of the *KCNJ11* E23K polymorphism with T2D was evaluated by calculating a pooled OR and 95% CI for allele contrast (K vs. E allele), heterozygous (KE vs. EE) and homozygote (KK vs. EE). Then, we examined the association between the polymorphisms and T2D risk using dominant and recessive genetic models. The standard Q-statistic test was performed to evaluate whether the variation between studies was due to heterogeneity or due to chance [7]. ORs were calculated according to the method of DerSimonian and Laird, and 95% CI was constructed by Woolf's method [8], [9]. In addition, subgroup analysis was used to investigate potential sources of heterogeneity by stratified meta-analyses based on ethnic group, sample size (No. cases ≥1000 or <1000) and mean BMI of cases (<25, 25∼30, or >30). Ethnic group was defined as East Asians, Caucasians (e.g., people of European origin), Indians and others (e.g., African American, Jews, and Arabian). Subsequently, ethnicity, sample size, BMI, age and sex were analyzed as covariates in meta-regression to further investigate potential sources of heterogeneity. For family-based association studies, the transmission disequilibrium test (TDT) was used to analyze effect size of the polymorphism. In general, the OR was calculated from the ratio of transmitted alleles to non-transmitted alleles from heterozygous parents to affected offspring [10], [11]. Combined effect size from both case–control and family-based association studies were calculated according to the method described previously by Lohmueller et al [12]. The Z-test was used to determine the significance of overall OR.

We calculated the sample size required for 80% power with the summary OR estimated from each ethnic populations, assuming an equal number of cases and controls, risk allele frequency (RAF) in controls estimated from different ethnicity. Furthermore, population attributable risk (PAR) was calculated to get a comprehensive view of the impact of the E23K variant on T2D at population level. PAR was calculated by the following formula: (OR-1)/OR * risk allele frequency [13].

Egger's test and funnel plots were used to assess small studies effects [14]. Sensitivity analysis was performed by excluding one study at a time to assess the stability of the results. The type I error rate was set at 0.05 for two-sided analysis. All of the calculations were performed using the STATA 10.0 (STATA Corporation, College Station, TX) and SAS (version 9.1; SAS Institute, Cary, NC).

## Results

### Characteristics of included studies

The literature search yielded 159 studies using keywords listed above. Figure S1 shows the literature search and selection process for eligible studies (Figure S1). Finally, a total of 48 studies including 56,349 cases, 81,800 controls and 483 family trios, were retrieved based on the search criteria for T2D susceptibility related to the *KCNJ11* E23K polymorphism [15]–[62]. In addition, almost all studies indicated that the distribution of genotype frequencies among the control groups were consistent with HWE. The detailed characteristics of all the included studies of this meta-analysis were summarized in Table 1.

**Table 1:** Characteristics of the studies included in the meta-analysis.

Study	Year	Ethnicity	Diagnostic criteria	Study design	No. of cases	No. of controls	MAF in cases/controls	Genotyping method	P _HWE_
Sakura [15]	1996	Caucasian	T2D patients	Population based-study	133	82	0.30/0.30	PCR-SSCP	0.05
Inoue [16]	1997	Caucasian	T2D patients	Population based-study	291	164	0.34/0.34	PCR-RFLP	>0.05
Hani [17]	1998	Caucasian	T2D patients	Population based-study	191	114	0.49/0.37	PCR-SSCP	0.95
Altshuler [18]	2000	Caucasian	T2D per WHO criteria	Family based-study	333 trios	/	/	SBE-FRET, SBE-FP	/
Yamada [19]	2001	East Asian	T2D per WHO criteria	Population based-study	103	73	0.39/0.34	PCR-SSCP	0.20
Gloyn [20]	2001	Caucasian	T2D patients	Population based-study	360	307	0.40/0.36	PCR-SSCP	0.09
Florez [21]	2004	Caucasian	T2D per WHO criteria	Population based-study	1077	1077	0.47/0.61	Flight mass spectroscopy	0.71
Barroso [22]	2003	Caucasian	T2D patients	Population based-study	499	494	0.38/0.34	FP-TDI	0.82
Gloyn [23]	2003	Caucasian	T2D per WHO criteria	Population based-study, Family based-study	854, 150 trios	1182	0.41/0.34	PCR-RFLP	0.53
Hansen [24]	2005	Caucasian	T2D per WHO criteria	Population based-study	1164	4733	0.40/0.36	PCR-RFLP, LNA	0.52
van Dam [25]	2005	Caucasian	T2D per WHO criteria	Population based-study	323	296	0.41/0.36	PCR-RFLP	0.56
Yokoi [26]	2006	East Asian	T2D per WHO criteria	Population based-study	1590	1244	0.38/0.37	MassARRAY	0.64
Liu [27]	2006	East Asian	T2D per WHO criteria	Population based-study	502	501	0.43/0.38	Sequencing	>0.05
Weedon [28]	2006	Caucasian	T2D per WHO criteria	Population based-study	2332	3592	0.38/0.35	TaqMan	>0.05
Sale [29]	2007	Other	T2D patients	Population based-study	572	587	0.06/0.07	MassARRAY	0.22
Koo [30]	2007	East Asian	T2D per WHO criteria	Population based-study	758	630	0.44/0.38	TaqMan	0.05
Sakamoto [31]	2007	East Asian	T2D per WHO criteria	Population based-study	906	889	0.39/0.34	TaqMan	0.72
Saxena [32]	2007	Caucasian	T2D per WHO criteria	Population based-study	5065	5785	0.49/0.47	Affymetrix GeneChip, MassARRAY	>0.05
Vaxillaire [33]	2007	Caucasian	T2D per ADA criteria	Population based-study	287	2684	0.41/0.39	TaqMan	0.68
Scott [34]	2007	Caucasian	T2D per WHO criteria	Population based-study	2295	2363	0.49/0.46	Illumina GeneChip, MassARRAY	0.72
Willer [35]	2007	Caucasian	T2D per WHO criteria	Population based-study	1087	953	0.49/0.44	MassARRAY	0.32
Qi [36]	2007	Caucasian	T2D patients	Population based-study	682	1078	0.40/0.35	TaqMan	0.38
Cejková [37]	2007	Caucasian	T2D per WHO criteria	Population based-study	172	113	0.37/0.37	PCR-RFLP	0.26
Doi [38]	2007	East Asian	T2D per WHO criteria	Population based-study	550	2322	0.39/0.34	TaqMan	0.46
Lyssenko [39]	2008	Caucasian	T2D per WHO criteria	Population based-study	2201	16034	0.41/0.40	TaqMan	>0.05
Alsmadi [40]	2008	Other	T2D per ADA criteria	Population based-study	550	335	0.21/0.14	TaqMan	0.40
Takeuchi [41]	2008	East Asian	T2D per WHO criteria	Population based-study	7954	8809	0.38/0.35	Illumina GeneChip, MassARRAY, TaqMan	0.91
Peng [42]	2008	East Asian	T2D per ADA criteria	Population based-study	275	168	0.69/0.57	PCR-RFLP	>0.05
Bronstein [43]	2008	Other	T2D patients	Population based-study	1131	1147	0.36/0.61	KASPar	0.58
Sanghera [44]	2008	Indian	T2D per ADA criteria	Population based-study	532	374	0.34/0.38	TaqMan	0.45
Cauchi [45]	2008	Caucasian	T2D per WHO criteria	Population based-study	2734	4234	0.37/0.37	TaqMan	0.69
Ezzidi [46]	2009	Other	T2D per ADA criteria	Population based-study	805	503	0.32/0.29	TaqMan	0.56
Zhou [47]	2009	East Asian	T2D per WHO criteria	Population based-study	1848	1910	0.41/0.39	TaqMan	0.39
Chistiakov [48]	2009	Caucasian	T2D per WHO criteria	Population based-study	129	117	0.50/0.39	PCR-RFLP	>0.05
Wang [49]	2009	East Asian	T2D per WHO criteria	Population based-study	396	387	0.46/0.37	SNapShot	0.46
Tabara [50]	2009	East Asian	T2D per ADA criteria	Population based-study	484	397	0.41/0.37	TaqMan	0.30
Thorsby [51]	2009	Caucasian	T2D patients	Population based-study	750	1879	0.41/0.41	PCR-RFLP	0.18
Hu [52]	2009	East Asian	T2D per WHO criteria	Population based-study	1849	1785	0.42/0.39	MassARRAY	>0.05
Yamauchi [53]	2010	East Asian	T2D per WHO criteria	Population based-study	4470	3071	0.38/0.37	Illumina GeneChip	>0.05
Neuman [54]	2010	Other	T2D patients	Population based-study	573	843	0.37/0.36	Pyrosequencing	0.22
Chauhan [55]	2010	Indian	T2D per WHO criteria	Population based-study	2434	2403	0.39/0.32	Golden Gate assay	0.41
Gupta [56]	2010	Indian	T2D per WHO criteria	Population based-study	209	179	0.40/0.47	Sequencing	0.12
Wen [57]	2010	East Asian	T2D per WHO criteria	Population based-study	1165	1135	0.41/0.40	MassARRAY	0.10
Rees [58]	2011	Indian	T2D per WHO criteria	Population based-study	1663	1567	0.38/0.38	TaqMan	0.13
Chavali [59]	2011	Indian	T2D per WHO criteria	Population based-study	1017	1006	0.39/0.35	Golden Gate assay	>0.05
Cheung [60]	2011	Chinese	T2D per WHO criteria	Population based-study	198	1185	0.33/0.33	TaqMan	0.41
Gamboa-Meléndez [61]	2012	Other	T2D per ADA criteria	Population based-study	1027	990	0.40/0.37	KASPAR	>0.05
Gonen [62]	2012	Other	T2D per ADA criteria	Population based-study	162	79	0.34/0.30	PCR-SSCP	NA

WHO: World health organization, ADA: American diabetes association, MAF: minor allele frequency, LNA: locked nucleic acid assay, FP-TDI: fluorescence polarization template-directed incorporation. SBE-FRET: single-base extension with fluorescence resonance energy transfer; SBE-FP: single-base extension with fluorescence polarization.

### Meta-analysis results

**Figure 1:**
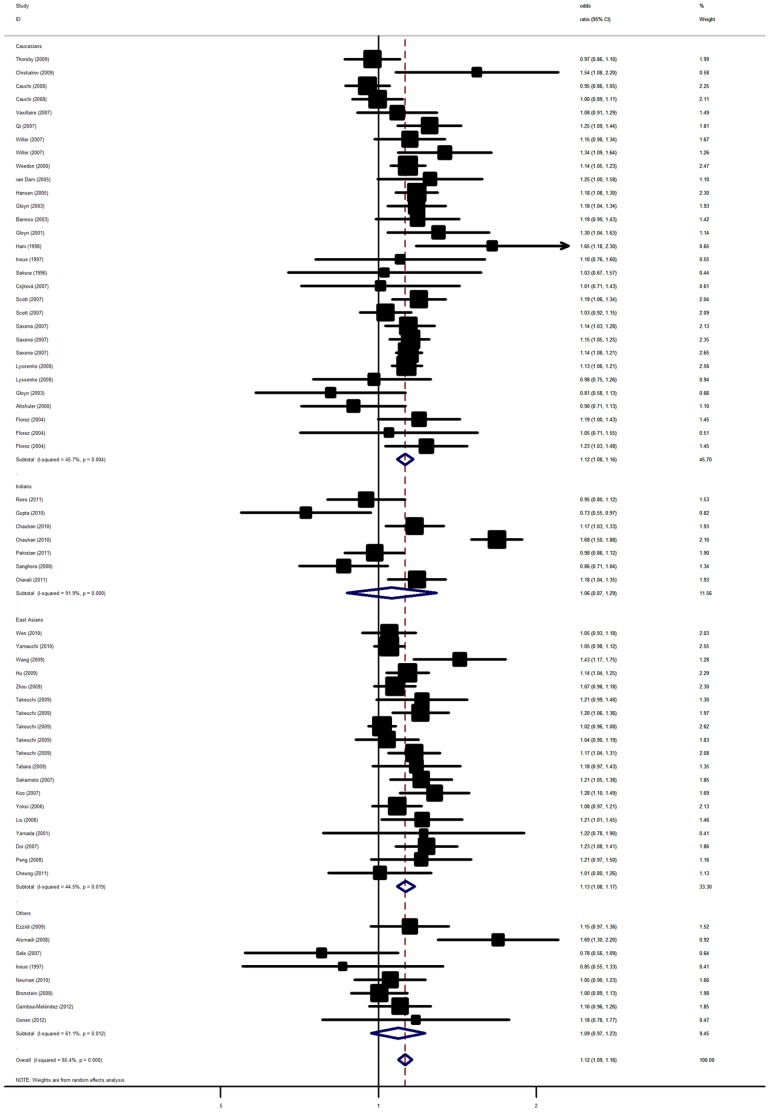
Forest plot from the meta-analysis of T2D risk and *KCNJ11* rs5219 polymorphism using random-effects model.

Overall, significant associations between *KCNJ11* E23K polymorphism and T2D were detected when all the eligible studies were pooled into the meta-analysis (Table 2). The overall result showed that the 23K allele of rs5219 polymorphism was significantly associated with elevated T2D risk with per-allele OR of 1.12 (95% CI: 1.08–1.17, *P*<10^−5^; Figure 1). Significant increased T2D risks were also detected for heterozygous (OR = 1.09, 95% CI: 1.03–1.14, *P*<10^−5^) and homozygous (OR = 1.26, 95% CI: 1.17–1.35, *P*<10^−5^) when compared with wild type homozygous. Similar results still maintained using dominant and recessive genetic models (Table S1). When studies were stratified for ethnic populations, significant associations were also observed among East Asian and Caucasian populations with per-allele OR of 1.13 (95% CI: 1.08–1.17, *P*<10^−5^) and of 1.12 (95% CI: 1.08–1.16, *P*<10^−5^) respectively. Significantly increased risks were also found for heterozygous and homozygous (Table 2). However, no such association was detected in Indian and other ethnic populations in all genetic models. In the subgroup analysis by sample size, significant associations were also observed for both large and small studies in all genetic models. When stratified by mean BMI of cases, statistically significant results were also observed for T2D cases with different BMI (Table 2). For two family-based association studies including a total of 483 family trios, we failed to detect statistically significant evidence for the risk 23K allele over-transmission from heterozygous parents to their T2D offspring (pooled OR_TDT_ = 0.87, 95% CI: 0.72–1.05; *P* = 0.14).

**Table 2:** Results of meta-analysis for *KCNJ11* E23K polymorphism and T2D risk.

Sub-group analysis	No. of studies	K allele				Heterozygous				Homozygous			
		OR (95%CI)	*P*(Z)	*P*(Q)^a^	*P*(Q)^b^	OR (95%CI)	*P*(Z)	*P*(Q)^a^	*P*(Q)^b^	OR (95%CI)	*P*(Z)	*P*(Q)^a^	*P*(Q)^b^
Ethnicity					0.10				0.06				0.01
Caucasians	22	1.12 (1.08–1.16)	<10^−5^	0.001		1.09 (1.06–1.13)	<10^−5^	0.13		1.33 (1.18–1.50)	<10^−5^	0.0008	
East Asians	14	1.13 (1.08–1.17)	<10^−5^	0.02		1.13 (1.05–1.22)	0.0009	0.02		1.30 (1.18–1.42)	<10^−5^	0.06	
Indians	5	1.06 (0.87–1.29)	0.56	<10^−5^		1.00 (0.82–1.23)	0.98	0.01		0.90 (0.69–1.18)	0.45	0.02	
Others	7	1.09 (0.97–1.23)	0.15	0.01		0.98 (0.76–1.28)	0.91	<10^−4^		1.19 (1.00–1.43)	0.05	0.31	
Sample size					0.32				0.14				0.04
Large	22	1.12 (1.08–1.17)	<10^−5^	<10^−5^		1.12 (1.10–1.15)	<10^−4^	0.12		1.17 (1.07–1.28)	0.007	0.02	
Small	26	1.13 (1.07–1.18)	<10^−5^	<10^−5^		1.10 (1.02–1.19)	0.009	0.002		1.32 (1.20–1.46)	<10^−5^	0.0007	
Mean BMI of cases					0.37				0.20				0.03
<25	12	1.15 (1.11–1.21)	<10^−5^	0.15		1.15 (1.08–1.22)	<10^−5^	0.21		1.40 (1.28–1.55)	<10^−5^	0.74	
25∼30	25	1.12 (1.06–1.19)	<10^−4^	<10^−4^		1.13 (1.06–1.21)	<10^−4^	0.004		1.18 (1.03–1.34)	0.008	<10^−4^	
>30	6	1.10 (1.03–1.18)	0.008	0.07		1.11 (1.04–1.19)	0.001	0.09		1.16 (1.01–1.33)	0.02	0.10	
Total	48	1.12 (1.09–1.16)	<10^−5^	<10^−5^		1.09 (1.03–1.14)	<10^−5^	0.0001		1.26 (1.17–1.35)	<10^−5^	<10^−5^	

*P*(Z): Z test used to determine the significance of the overall OR.

*P*(Q)^a^: Cochran's chi-square Q statistic test used to assess the heterogeneity in subgroups.

*P*(Q)^b^: Cochran's chi-square Q statistic test used to assess the heterogeneity between subgroups.

Significant heterogeneity was found among the 46 included studies (*P*<10^−5^). Hence, meta-regression was further conducted to investigate the source of heterogeneity. In meta-regression analysis, ethnicity (*P* = 0.79), sample size (*P* = 0.61), mean age (*P* = 0.36) of cases and controls (*P* = 0.61), gender distribution in cases (*P* = 0.96) and controls (*P* = 0.30) did not explain a large part of the heterogeneity among the individual study. By contrast, mean BMI (*P* = 0.03) explained about 11% of the heterogeneity.

**Figure 2:**
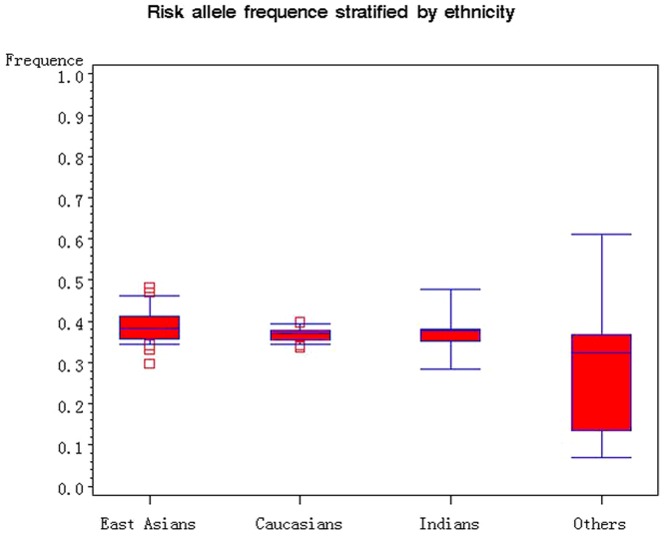
Frequencies of the 23K allele of *KCNJ11* rs5219 polymorphism among controls stratified by ethnicity. The “□” represent outlier.

The 23K allele frequency of the rs5219 polymorphism varies in the control groups across different ethnic populations, ranging from 0.07 to 0.61 (Figure 2). In Caucasian controls, the K allele frequency was 0.40 (95% CI: 0.37–0.42), which was higher than that of East Asian controls (0.36; 95% CI: 0.34–0.38), Indian controls (0.34; 95% CI: 0.27–0.41). 2500 and 2200 case-control pairs will be required for 80% power to detect the risk allele among Caucasian and East Asian population respectively. The population attributable risk (PAR) of T2D related to E23K polymorphism was 4.6% overall, 4.4% for Caucasians and 4.5% for East Asians.

### Sensitivity analyses and publication bias

The results of sensitivity analysis confirmed the significant associations of the *KCNJ11* E23K polymorphism with T2D risk, and no single study influenced the overall OR qualitatively (Figure S2). The Egger's test and funnel plots indicated no publication bias for the association of *KCNJ11* E23K polymorphism and T2D (Figure S3; Egger test, *P*>0.05).

## Discussion

Limited statistical power of relative small sample size is a common problem in genetic association for individual T2D studies. Therefore, sufficient sample power is necessary in deciphering genetic architecture of T2D, but it is sometimes very difficult for a single study to collect enough amounts of data to reach a reliable conclusion. By pooling of data from individual association studies, meta-analysis is an effective approach of increasing the sample size under investigation, thus enhancing the statistical power for the estimation of genetic effects.

Our results indicated that the rs5219 polymorphism of *KCNJ11* is a risk factor for developing T2D. In the subgroup analyses by ethnicity, we found the rs5219 polymorphism was associated with T2D among East Asians and Caucasians, but not Indians or other ethnic populations. Of note, different ethnic populations were pooled in the other ethnic group and only a few studies were available in the subgroup, so the result must be interpreted with caution. There are several other possible reasons which may account for such differences. First, T2D is a complex disease and different genetic backgrounds may cause the discrepancy since the distributions of the risk-association alleles in *KCNJ11* were different between various ethnicities. The K allele frequency of the rs5219 polymorphism was ∼36%, ∼40%, and ∼34%, among East Asians, Indians and Caucasians populations, respectively. Such result could also be due different linkage disequilibrium (LD) pattern of the polymorphism and nearby causal variant among different ethnic populations. Moreover, inter-individual difference like age, sex, dietary intake of nutrients, in addition to phenotype heterogeneity, such as years from onset and severity of the disease may also explain the discrepancy. Furthermore, study design and/or small sample size or some environmental factors may also affect the results. Therefore, more studies are needed to further validate the effect of the polymorphism on T2D risk among difference ethnic populations.

The *KCNJ11* gene has attracted considerable attention as a promising candidate for T2D based on its position and its function as a key factor in the regulation of glucose-induced insulin secretion, since normoglycemic lysine carriers are shown to consistently display a defect in insulin secretion [21], [63], [64]. Functional studies suggested that the KK genotype might induce a critical inhibition of glucose-induced insulin release from pancreatic β-cells [65]. Furthermore, the *KCNJ11* E23K variant was found to be associated with glucose intolerance and conversion from impaired glucose tolerance to T2D among Caucasians [66], [67]. Previous studies indicated that the E23K variant is functional by affecting *in vitro* properties of KATP channel via increasing the threshold of ATP concentration for insulin secretion [65], [68].

The distribution of the E23K variant in controls across various studies showed global variation (Figure 2), suggesting the possibility of population stratification. However, empirical evidence indicated that well-designed population-based association studies can keep the effects of population stratification to a minimum [69]. Almost all the population-based genetic association studies included in the present meta-analysis were well-designed by recruiting cases and controls from the same geographic region and ethnicity, which may help to reduce the effects of population stratification. Moreover, the effects of potential population stratification in any individual study may be in a random direction, so that one individual study with a small amount of stratification should have very limited effect on the overall results [70]. By combining results from TDT studies, which are robust to potential population stratification, we failed to detect an over-transmission of the 23K allele from heterozygous parents to their T2D offspring. Given the small number of studies and relative small sample size, the combined results from TDT studies should be interpreted with caution.

Power analysis revealed a hint in design for future association studies. So far, most association studies have included at the most several hundred subjects and results from these studies should be treated with caution as for limited statistic power to reach a reliable conclusion. According to our power analysis, powerful association studies on the E23K polymorphism and T2D risk may need several thousand individuals. Compared with candidate gene approach, genome wide association study (GWAS) with large sample size and unbiased to genomic structure is a powerful approach in susceptible gene identification for T2D. Recently, Tsai et al. [71] reported a two-stage genome-wide association (GWA) conducted in Chinese and identified *KCNJ11* as a risk region in T2D susceptibility which was in line with the results the present meta-analysis.

In comparison with the previous published meta-analysis [63], [66], [72]–[74], the current study included more than ten times as many cases as the earlier meta-analysis. In addition, we assessed the effect of risk allele and T2D using various genetic models and reached consistent results. Furthermore, we systematically explored potential sources of heterogeneity across studies and the possibility of publication bias. In addition to combine those newly published data, the present study also statistically joined population-based and family-based genetic association studies into a single meta-analysis, which allowed us to enhance the power of the meta-analyses and also establish a comprehensive picture of the relationship between *KCNJ11* and genetic susceptibility to T2D.

Heterogeneity among pooled studies is a frequently encountered issue in meta-analyses. Of note, our meta-analyses joined population bases association studies and family based association studies, which could enhance the heterogeneity. Hence, we performed subgroup analysis and meta-regression to identify potential source of between-study heterogeneity. However, they revealed that only BMI of controls could explain a small part of significant heterogeneity between studies. There may be a number of possible underlying reasons. Firstly, results from case–control studies may differ because of ethnic diversity (e.g., variation in allele frequencies) and geographic variation. Secondly, variations in methods of sample ascertainment and diagnosis may also contribute to such inconsistence. Thirdly, environmental factors, such as alcohol drinking, smoking behavior, obesity might also result in variability between studies.

To conclude, this may be the most comprehensive meta-analysis of *KCNJ11* and T2D. Our results suggest a modest but statistically significant effect of the 23K allele of rs5219 in susceptibility to T2D, particularly in East Asians and Caucasians. More work will be required for future association studies, especially those which are properly powered, effectively control for confounding factors, employing family-based design. Moreover, gene–environment and gene–gene interactions should also be taking into consideration for future studies.

## Supporting Information

Checklist S1 (DOC)

Figure S1 **Flow chart of literature search for studies examining** ***KCNJ11*** **rs5219 polymorphism and risk of T2D.**
(TIF)

Figure S2 **Result of sensitivity analyses.**
(TIF)

Figure S3 **Begg's funnel plot of rs5219 polymorphism and T2D risk.**
(TIF)

Table S1 **Results of meta-analysis for** ***KCNJ11*** **E23K polymorphism and T2D risk using dominant and recessive genetic models.**
(DOCX)
