# Supplementary figures and images for: Quantitative Assessment of the Effect of KCNJ11 Gene Polymorphism on the Risk of Type 2 Diabetes
Source: PLoS One. 2014 Apr 7;9(4):e93961. doi: 10.1371/journal.pone.0093961 (PMC3977990; doi:10.1371/journal.pone.0093961)

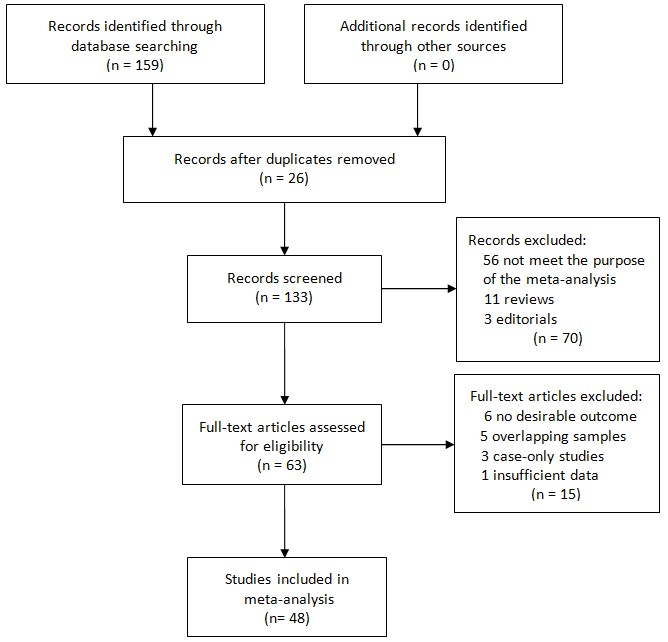

Supplement: Figure S1 — Flow chart of literature search for studies examining KCNJ11 rs5219 polymorphism and risk of T2D. (TIF) [file pone.0093961.s002.tif]

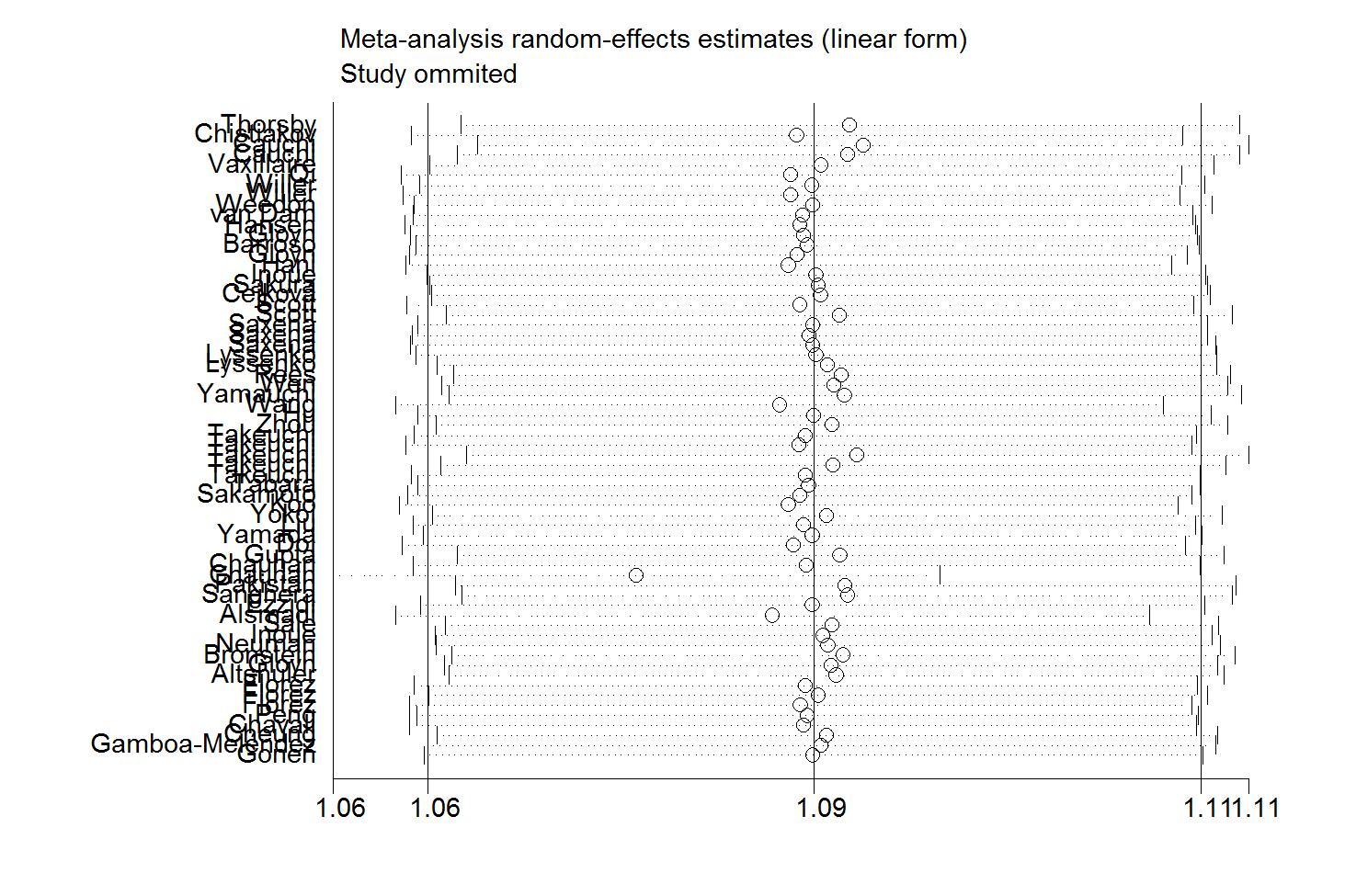

Supplement: Figure S2 — Result of sensitivity analyses. (TIF) [file pone.0093961.s003.tif]

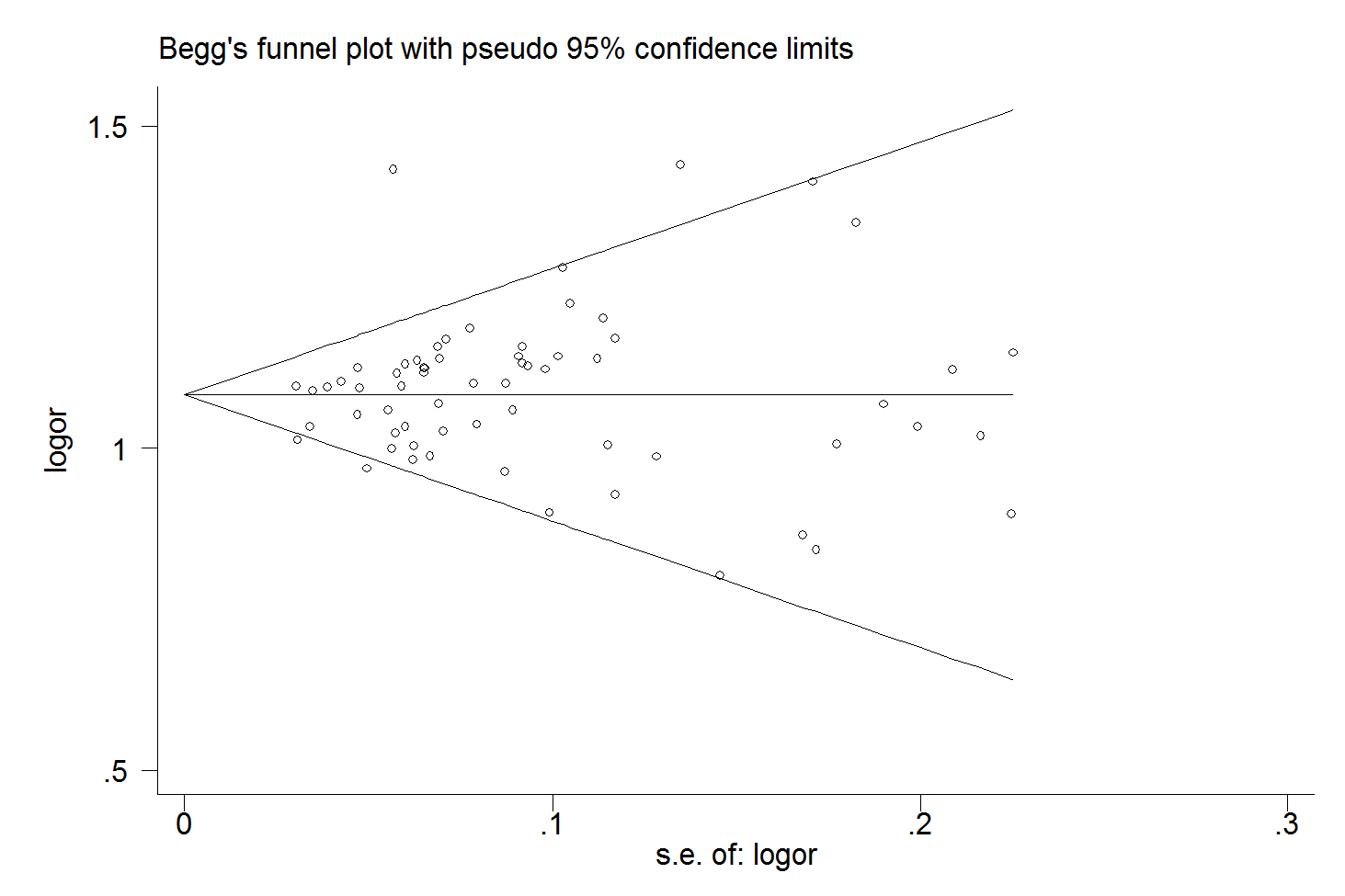

Supplement: Figure S3 — Begg's funnel plot of rs5219 polymorphism and T2D risk. (TIF) [file pone.0093961.s004.tif]
