# Supplementary material for: Quantitative Assessment of the Effect of KCNJ11 Gene Polymorphism on the Risk of Type 2 Diabetes
Source: PLoS One. 2014 Apr 7;9(4):e93961. doi: 10.1371/journal.pone.0093961 (PMC3977990; doi:10.1371/journal.pone.0093961)
Supplement: Table S1 — Results of meta-analysis for KCNJ11 E23K polymorphism and T2D risk using dominant and recessive genetic models. (DOCX) [file pone.0093961.s005.docx]

| Sub-group analysis | No. of studies | Dominant model | | | | Recessive model | | | |
| --- | --- | --- | --- | --- | --- | --- | --- | --- | --- |
|  |  | OR (95%CI) | *P*(Z) | *P*(Q)^a^ | *P*(Q)^b^ | OR (95%CI) | *P*(Z) | *P*(Q)^a^ | *P*(Q)^b^ |
| Ethnicity |  |  |  |  | 0.22 |  |  |  | 0.01 |
| Caucasians | 22 | 1.10 (1.07-1.15) | <10^-5^ | 0.008 |  | 1.30 (1.16-1.43) | <10^-5^ | <10^-4^ |  |
| East Asians | 14 | 1.12 (1.07-1.19) | <10^-5^ | 0.05 |  | 1.28 (1.17-1.41) | <10^-5^ | 0.02 |  |
| Indians | 5 | 1.03 (0.92-1.16) | 0.42 | <10^-4^ |  | 1.01 (0.74-1.57) | 0.61 | 0.007 |  |
| Others | 7 | 1.05 (0.95-1.20) | 0.27 | 0.003 |  | 1.08 (0.99-1.27) | 0.07 | 0.22 |  |
| Sample size |  |  |  |  | 0.55 |  |  |  | 0.06 |
| Large | 22 | 1.12 (1.09-1.16) | <10^-5^ | <10^-5^ |  | 1.26 (1.15-1.39) | <10^-5^ | <10^-4^ |  |
| Small | 26 | 1.11 (1.06-1.20) | <10^-5^ | <10^-5^ |  | 1.28 (1.20-1.51) | <10^-5^ | <10^-4^ |  |
| Mean BMI of cases |  |  |  |  | 0.08 |  |  |  | 0.01 |
| < 25 | 12 | 1.15 (1.10-1.20) | <10^-5^ | 0.24 |  | 1.30 (1.20-1.44) | <10^-5^ | 0.30 |  |
| 25~30 | 25 | 1.11 (1.05-1.20) | <10^-4^ | <10^-4^ |  | 1.20 (1.09-1.38) | <10^-4^ | <10^-5^ |  |
| > 30 | 6 | 1.11 (1.04-1.18) | 0.003 | 0.05 |  | 1.25 (1.14-1.39) | <10^-5^ | 0.003 |  |
| Total | 48 | 1.11 (1.08-1.17) | <10^-5^ | <10^-5^ |  | 1.27 (1.17-1.36) | <10^-5^ | <10^-5^ |  |

**Table S1** Results of meta-analysis for *KCNJ11* E23K polymorphism and T2D risk using dominant and recessive genetic models.

*P*(Z): Z test used to determine the significance of the overall OR.

*P*(Q)^a^: Cochran's chi-square Q statistic test used to assess the heterogeneity in subgroups.

*P*(Q)^b^: Cochran's chi-square Q statistic test used to assess the heterogeneity between subgroups.
